# Healthcare bricolage in Europe’s superdiverse neighbourhoods: a mixed methods study

**DOI:** 10.1186/s12889-019-7709-x

**Published:** 2019-10-22

**Authors:** Jenny Phillimore, Tilman Brand, Hannah Bradby, Beatriz Padilla

**Affiliations:** 1 Institute for Research into Superdiversity (IRiS), School of Social Policy, University of Birmingham, Edgbaston, Birmingham, B15 2TT UK; 2 Leibniz Institute for Prevention Research and Epidemiology – BIPS, Achterstraße 30, D-28359 Bremen, Germany; 3 Sociology Department, Uppsala University, Box 624, Se-751 26 Uppsala, Sweden; 4 University of South Florida, Fowler Avenue, Tampa, FL 33620 USA; 5 Instituto Universitario de Lisboa (ISCTE-IUL), Lisbon, Portugal

**Keywords:** Superdiversity, Bricolage, Public healthcare, Healthcare seeking, Trust, Agency, Health literacy

## Abstract

**Background:**

Studies of the relationship between diverse populations, healthcare access and health outcomes have been dominated by approaches focusing on ethno-national groups or specific healthcare sectors. Healthcare bricolage conceptualises the processes by which individuals use multiple resources to address health concerns. It is relevant in superdiverse neighbourhoods with complex populations. This paper is original in its application of mixed methods to examine the extent to which, and the reasons why, individuals engage in healthcare bricolage.

**Methods:**

The study utilized a parallel sequential methodology. Eight superdiverse neighbourhoods were selected, two in each of Bremen, Birmingham, Lisbon and Uppsala. Ethnographic research scoping the nature of each healthcare ecosystem was followed by 160 interviews (20 each neighbourhood) with a maximum variation sample of residents undertaken October 2015 to December 2016. Interviewees were asked to recall a health concern and describe actions taken to attempt resolution. Data was coded with a MAXQDA codebook checked for inter-coder reliability. Interview findings enabled identification of five types of bricolage, the nature of healthcare resources utilised and the factors which influenced residents’ tactics. Results were used to design a household survey using new questions and validated epidemiological instruments implemented January to October 2017. Respondents were identified using random address files and interviewed in person or by telephone. Multinomal logistic regressions were used to estimate the effect of changing the values of determinants on the probability of observing an outcome.

**Results:**

Age, gender, level of education, migration background and extent of functional limitation were associated with bricolage tactics. Individuals demonstrating high levels of agency were more likely than those with low levels to engage in bricolage. Residents with high levels of trust in physicians were less likely to bricolage than those with lower levels of trust. Levels of health literacy showed no significant effects.

**Conclusions:**

The nature and severity of health concern, trust in physicians and agency shaped residents’ bricolage tactics. The concept of bricolage enabled us to make visible the actions and resources utilised around public healthcare systems that would otherwise remain outwith healthcare access research. Actions were frequently undertaken via networks offering insights into healthcare-seeking behaviour.

## Background

Studies of the relationship between diverse populations, access to healthcare and health outcomes have been dominated by approaches focusing on ethno-national groups or specific healthcare sectors. Healthcare bricolage conceptualises the processes by which individuals use multiple resources to address their health concerns. It has particular application in superdiverse areas where there are no dominant ethno-national groups and populations are heterogenous both within and across groups. This paper is the first to examine empirically the extent to which, and the reasons why, individuals engage in healthcare bricolage to address their health concerns. It utilizes data from the UPWEB study, which employs a mixed methods approach comprising in-depth interviews followed by a household survey in superdiverse neighbourhoods in four European cities: Birmingham, Bremen, Lisbon and Uppsala.

The paper focuses on three factors that have potential to shape individuals’ bricolage tactics: levels of health literacy; trust in physicians and individual agency. It finds that trust and agency have considerable explanatory potential but that the importance of health literacy is not supported by the survey data. The paper begins by describing the emergence of superdiversity as a demographic phenomenon before briefly outlining the shortcomings of more conventional approaches to researching diversity and healthcare. The notion of healthcare bricolage is introduced and its potential to explore individual approaches to addressing health concerns in superdiverse neighbourhoods outlined. Health literacy, trust and agency are introduced as factors with potential to explain the reasons why individuals engage in bricolage. Methods, including the relationship between interviews and survey instruments, are outlined before we move to describe the extent of bricolage in each city, the characteristics of the individuals who bricolage and the reasons why they may bricolage. The paper ends with a discussion of the findings and the usefulness of healthcare bricolage as a concept.

### The emergence of superdiversity

The term superdiversity, coined by Stephen Vertovec in 2004, has been widely adopted across Europe to signal the major demographic shift underway since the 1990s. This move from the predominance of “old” migration based on post-colonial relations and/or bi-lateral labour agreements, to “new” migration, whereby people arrive from many different countries, acknowledging the intra-group heterogeneity of migrant and minority populations. The so-called diversification of diversity whereby individuals’ experiences and needs are shaped by multiple factors including immigration status, income levels, faiths, educational attainment, ethnicities, rights and entitlements and spatial distributions have resulted in increased levels of heterogeneity [1]. While the term superdiversity has received considerable criticism, particularly around its theoretical limitations, it has been widely adopted as a demographic descriptor. Policymakers and service providers have utilised the term across Northern Europe as a means to describe the intensified diversity to which social, health and welfare services must respond. Superdiverse neighbourhoods change quickly and they often lack sufficient numbers from a particular country of origin or language group, around which to provide specialised or tailored health and welfare services, as has been the practice where a multicultural approach to service provision has been the norm [2]. Demographic complexity and fast-paced change present challenges around how healthcare needs are identified, services commissioned and improved through evaluation and research. Difficulties facilitating equitable access to services exacerbates long-term failures in resolving inequalities of outcome and inequity of access in areas with high levels of diversity [3].

### Diversity and access to healthcare

The post-World War II development of socialised healthcare systems in Europe focussed on universal provision through two main models: National Health Services or insurance-based health systems, both designed to meet the needs, as assessed by professionals, of predominantly white populations. As Europe became more diverse different categorisations were introduced to enable monitoring of migrants’ and their descendants’ access to services, morbidity, mortality and behaviours. However, the lack of a coordinated system across Europe has worked against cross-national research that may have enabled identification of effective provision [4]. The functionality of ethno-national categorisations in the context of the ‘diversification of diversity’ [1] is limited. There is a need for approaches that can explore the challenges faced by increasingly mobile, fragmented and complex superdiverse populations in accessing healthcare [5]. Further it is necessary to shift beyond research that focuses on access to service provision by sector, examining the role of the state [6], the voluntary [7] and private sectors [8] separately, and to include informal provision [9], internet provision [10] and transnational strategies [11]. Elsewhere we introduce the concept of healthcare bricolage to move beyond existing approaches to researching access to healthcare in diverse communities that silo provision, failing to consider healthcare systems as ecosystems comprising multiple providers [12] and how individuals may access different sectors or adopt multiple approaches either simultaneously or consecutively.

Building on the work of Levi-Strauss [13], De Certeau [14], and Deleuze and Guattari [15] we invoke the concept of bricolage showing its potential to “*encapsulate actions which involve individuals undertaking practices to augment existing provision, as an alternative to existing provision, or as a necessity go beyond existing provision*” [16]. We show how bricolage can be used as a response to scarce healthcare resources, a creative alternative to existing provision, and/or making use of available or reusing existing resources. As such we define healthcare bricolage as “*a creative mobilisation, use and re-use, of wide ranging resources, including multiple knowledges, ideas, materials and networks in order to address particular health concerns*” [16]. The concept of medical pluralism has enjoyed some reinvention with interest in the relationship between biomedicine and complementary systems in migration settings [8]. Medical pluralism considers ‘the existence in a single society of differentially designed and conceived medical systems’ [17] and so offers limited insight to transnational assemblages of practices and resources. Furthermore medical pluralism tends to privileging professionals’ perspectives [18], conceptualising other perspectives as modifying the biomedical and the professionally validated. Bricolage offers greater insight into the *how* and *why* of what people do to address health concerns, giving equal weight to all perspectives that are in play.

We suggest that superdiverse neighbourhoods which are often resource poor may offer both necessity and opportunity to bricolage. Such neighbourhoods house people from many different places offering a wide range of diverse resources including different belief systems, cultural backgrounds, languages, networks and levels of institutional awareness all of which offer potential to enable bricolage. Applied in superdiverse neighbourhoods where transnational connections are commonplace, the notion of bricolage can be used heuristically to make visible the ways in which people connect resources from across localities, the world, and different medical systems. In this paper, we investigate empirically the extent to which, and the reasons why, individuals engage in healthcare bricolage and explore how they address health concerns focusing on three possible explanations: health literacy; levels of trust in healthcare professionals and agency.

### Health literacy

The notion of health literacy has gained considerable traction in clinical care and public health [19]. It is generally used as measure of patients’ ability to comprehend and act on medical instructions [20]. Poor health literacy is argued to be common amongst minority ethnic groups and is seen as a factor contributing to the poor management of chronic conditions [20], and critical illness [21] and an explanatory factor in poor health outcomes [22]. More recently the concept has moved beyond its origins in educational literacy and been applied to the ability to understand health systems [23]. As Europe becomes increasingly diverse, health literacy is seen as particularly relevant for migrants who may be more familiar with healthcare institutional cultures in their country of emigration than the country of immigration [23]. Various scholars have shown how low levels of health literacy have associations with individuals’ knowledge about how to utilize systems to their full potential, for example how to access antenatal care [5] or their expectations of care [24]. Arguably, the extent to which people can understand public healthcare systems, and those systems meet their expectations, has the potential to affect their propensity to engage in healthcare bricolage. Unsuccessful or unsatisfactory engagement resulting from a lack of knowledge about how to navigate systems could prompt individuals to bricolage using other, more familiar resources including out-of-pocket services, transnational healthcare or complementary therapies.

### Trust

Trust in physician and medical institutions is a “*global attribute of treatment relationships, one that encompasses subsidiary features such as satisfaction, communication, competency, and privacy*” [25]. It is based on intrinsic and instrumental values that frame healthcare provider-patient relations in therapeutic encounters translating into patients’ willingness to seek care, and impacting upon health systems [26]. As a relational phenomenon, trust enhances cooperation, so it is central to good interactions between patient and provider [25, 26]. Trust can be vertical or horizontal, the first implies trust in institutions and the second in people [27]. The quality of encounters with healthcare professionals can develop or undermine trust and have a bearing on the long-term relationship between patient and individual clinicians or entire systems [26]. Trust has an emotional component, prominent in medical contexts, which is not always rational and objective and may arise as a coping mechanism for distress generated by illness, thereby playing out as a need to believe [25]. However there is clear evidence that trust in the healthcare system encourages people to use services [26], to accept treatments offered [25], to follow treatments as recommended [26, 28] and to disclose important information [29] while mistrust discourages people from seeking healthcare [30]. Health communication strategies are often based on an interactive exchange of information to improve access; trust plays a key role in this exchange, translating into more effective communication [31]. People base their trust on individual experiences and on social and cultural traditions, however superdiverse neighbourhoods, where different and conflicting traditions are prevalent, tend to be low-trust environments [31]. There are well-documented problems with communication between clinicians and migrant patients [32], knowledge and understanding of health services and limited institutional capacity to engage with individuals unfamiliar with languages, systems or cultures [33]. A low level of trust in healthcare providers has been identified as one reason why some migrants return home to access care [34] and important in relation to getting appropriate, fair and culturally competent mental [35] and physiological healthcare [36]. Thus, it is possible that there will be a relationship between the extent to which people living in superdiverse areas trust/mistrust health professionals and use bricolage as a strategy or coping mechanism. Those with lower levels of trust in public healthcare systems may be more inclined to use outside and/or alternative resources.

### Agency

Individual agency’s role in producing health and health inequalities in the context of social structure has a long history of theoretical and empirical investigation [37]. Agency is defined as the ability for people to deploy a range of causal powers [38]. People living in extremely poor circumstances have strategies to distance themselves from the negative aspects of their locality, which plays a role in the creation of health inequalities as well as resistance to their negative implications [39]. The ability to make the most of poor material circumstances (which is not the same as negating them) lies in social relationships, networks and a sense of control over life [40]. The extent to which agency can overcome poor material circumstances is not easy to establish but it is certainly relevant at the level of lay explanations of health and illness and for people’s dignity and ability to survive adversity. Thus, it is conceivable that individuals with high levels of agency might be inclined to bricolage in order to take control over the treatment of their health concern.

## Methods

The study utilized a parallel sequential mixed methods approach in which each phase of the research informed the next [41]. We selected one city in each of four different countries with four different welfare regime “ideal types” [42] (see Additional file 3: Table S1): Germany, Portugal, Sweden, and the UK. Each country was sufficiently different to allow comparison [43]. Ethical approval was obtained from the ethics committees of the involved universities and the regional ethics committee in Uppsala (ERN_14–1111 for lead university). The project was an interdisciplinary endeavour bringing together epidemiologists with medical anthropologists and sociologists, social policy analysts, geographers and political scientists.

### Qualitative research

The study began with an ethnographic phase wherein researchers walked two selected superdiverse neighbourhoods in each city observing how different healthcare resources were used while engaging in conversations about healthcare with residents and providers. Insights from observations were used to develop a semi-structured interview guide (see Additional file 1). Male and female academic researchers and trained community researchers interviewed some 160 residents across the four countries (for interviewee profile see Additional file 3: Table S2) between October 2015 and December 2016. Community researchers were recruited for their interpersonal and multi-lingual abilities and knowledge of local networks. Each country team trained community researchers using an adapted version of an accredited model developed by the UK team [41]. The community researchers were paired with academic researchers and together identified interviewees via networks, local organisations, and snowball sampling through street mapping and interaction with locals. Maximum variation sampling was used to ensure heterogeneity in terms of origin, age, gender, education levels, income, ethnic and linguistic backgrounds. This comparison-focused sampling approach selects cases to identify factors explaining similarities and differences [44]. Commonalities that emerge, despite many intersecting axes of difference, have increased validity because they do not result from sampling by pre-determined characteristics. Maximum variation was achieved through each country team completing a table containing columns with different characteristics as detailed above. As respondents were identified, details of the characteristics were cross-checked to examine levels of similarity or difference with previous respondents, and a decision made as to whether to interview the prospective respondent. Further as data collection progressed gaps in characteristics were identified and specific efforts made to identify respondents meeting those criteria. For example, one of the neighbourhoods in Birmingham was known to be a dispersal area for asylum seekers yet at near the end of the data collection no asylum seekers had been interviewed so the research team actively sought to access an asylum seeker respondent. Data saturation was reached once no new patterns in bricolage behaviours were identified.

Residents were interviewed for between 30 and 90 min in their preferred language after receiving a participant information form outlining the purpose of the project, being given the opportunity to ask questions about the study, informed how data would be utilised and then asked if they wanted to participate. Interviewees and interviewers signed consent forms stressing confidentiality and the option to withdraw from interviews at any time during the actual interview or to withdraw their data up to 30 days after the interview was completed. Names used in this paper are pseudonyms. Interviewers using a piloted interview guide asked people to recall a recent health concern and to describe actions taken from the emergence of symptoms until some resolution was reached (see Additional file 1). Participants were interviewed at the location of their choice which included cafes, homes and non-government organisation (NGO) premises.

Prompts such as “did you do anything else?” were used to support recall of the range of actions enabling a social constructivist approach to be implemented. All interviews were digitally recorded, transcribed and where necessary translated. Country leads checked transcripts to ensure quality, consistency and absence of bias. Data were coded collectively using a systematic thematic analysis approach [45] to inductively identify key issues. This involved interpretive code-and-retrieve methods wherein the data were read by the research team who collectively identified codes and engaged in interpretative thematic analysis. Coding commenced with a three-day workshop in which all team members worked collectively to devise codes based on their reading of transcripts from each country. The shared coding frame was then devised using MAXQDA software and consolidated by the Project Lead, Phillimore, and piloted by all researchers in each country team. Following the piloting the frame was revised by Phillimore and then implemented by the research fellow in each team. Coding was overseen by each country lead. The project lead sought to maximise inter-coder reliability across sites by using the units of meaning and negotiated agreement approaches outlined by Campbell et al. [46] which rely heavily upon precise definitions of codes and through ongoing discussion around “what code might this be” facilitated by Phillimore both via e-mail and videoconferencing across the entire coding period (3 months) .

### Bricolage tactics

Analysis of these semi-structured interviews was used to identify five types of bricolage tactics summarised below:

- No bricolage: respondents used only the NHS/ public healthcare system, i.e. all services and treatments were covered by their health insurance or publicly financed and for which they did not pay fully out-of-pocket using services as instructed without outside support, advice or guidance.
- Within-system bricolage: respondents used the NHS/ public healthcare system plus informal support from family and friends or information sources such as the internet or books and magazines to address the health concern.
- Added-to-system bricolage: respondents added advice, services or treatments that were not covered by the NHS/ public healthcare system including out-of-pocket services, alternative or complementary medicine or services from another country.
- Alternative: respondents did not use the NHS/ public healthcare system but used services not covered by the public system or informal and informational support.
- No resources used: respondents did not use any resources to address the health concern.

Our notion of healthcare bricolage is based upon the idea, emerging from both our interview findings and the literature, that use of the public healthcare system is the normative approach to addressing a health concern and that this “system” would be utilised unaided and un-adapted. The adaptions made to the “norm” are conceived of as bricolage since they involve both creativity and the use of additional resources as outlined in our discussion above. Clearly there are multiple approaches to bricolage, but we have categorised these into five recurring patterns. These move from “no bricolage” where the system is used unaided to “within system bricolage” where the creativity is used to adapt public healthcare resources and additional information, or guidance, is levered in from outside the system in order to make it accessible. Three other categories are identified. These are “alternative” wherein the public healthcare system is not used in any way, but other resources are used instead and “no resources” wherein respondents took no action of any kind and used no resources whatsoever to address their concern. Finally, “added to the system bricolage” describes a situation wherein respondents continue to use the public healthcare system but supplement it with additional actions which require resources and/or creativity. Analysis of semi-structured interview data also enabled us to identify the nature of resources utilised for bricolage and the factors which appeared to influence bricolage tactics.

We use the Oxford English Dictionary definition of tactics “*an action or strategy planned to achieve a specific end*” [47]. We then devised a household survey (see Additional file 2) using questions developed through the qualitative analysis together with existing validated epidemiological instruments to answer the following research questions:

- How extensive was bricolage in response to health concerns?
- What types of bricolage were adopted?
- What types of resident utilized the different kinds of bricolage?
- What factors shaped bricolage tactics?

### Household survey

Potential respondents were identified by using random address files of the respective neighbourhoods. Persons were eligible for participation if they were neighbourhood residents over 18 years old. The aim was to sample at least 300 individuals per neighbourhood. Since this was an explorative analysis, the sample size was not based on a power analysis. The fieldwork was undertaken between January and October 2017. Respondents were approached via invitation letters, phone calls and door-to-to visits. Response proportions were 53% in Birmingham, 15% in Bremen, 22% in Lisbon, and 14% in Uppsala. The interviews were conducted by multilingual staff either face-to-face or over the telephone. All participants provided written, or for telephone interviews verbal, informed consent.

As we were particularly interested in the bricolage tactics of residents living in superdiverse neighbourhoods, respondents were asked how long they had been resident in the neighbourhood and whether they experienced any health concerns while living there. Their health concern was recorded and they were asked which resources they had used to address that concern. To ensure confidentiality no names or addresses were recorded on the completed questionnaires. All data from across the study were stored on encrypted computers and only accessible to the research team.

### Factors related to bricolage tactics

#### Individual characteristics

Sociodemographic, migration and health-related variables were assessed to describe the characteristics of individuals utilizing different forms of bricolage. Sociodemographic variables included age, gender, education and employment status. Educational level was categorized into three groups according to the International Standard Classification of Education (ISCED; low 0–2; medium 3–4; high 5–6), and employment status was dichotomized into unemployed yes/no. Migration background was assessed based on the interviewee’s country of birth, that of their parents and/or grandparents. Foreign-born residents were coded as migrants; those with at least one parent born abroad were coded as second-generation migrants; and those with at least one grandparent born abroad were coded as third-generation migrants. Duration of residence in the survey country was assessed as well as self-rated proficiency in the country’s national language.

Language proficiency was rated on a 5-point scale and then dichotomized for analysis (good = good very good/ good; poor = fair/ poor/ very poor). A single-item self-rated health question was used as an indicator for general health status. The five response options were dichotomized into good (excellent/ very good/ good) and poor (fair/ poor). Furthermore, functional limitations associated with the respondent’s health concerns were assessed as an indicator of needing healthcare.

#### Health literacy, trust and individual agency

We assessed health literacy, trust in physicians and agency to examine the extent to which there was a relationship between these and the tendency to engage in bricolage. Health literacy was assessed using the 6-item short version of the European Health Literacy Survey Questionnaire (HLSEU-Q6 [48]). Internal consistency was good (α = 0.81). Mean scores from the 4-point scale were calculated and dichotomized into low (1.0–2.0) and medium/ high (> 2.04.0). Trust in physicians was assessed using a 4-item short version of the scale developed by Thom et al. [49]. The responses were transformed into a summary score ranging from 0 to 16. Internal consistency was acceptable (α = 0.73). Based on the distribution in our sample the scores were categorized into tertiles (low = 0–9; medium = 10–12; high = 13–16). In addition, based on the insights from the qualitative phase, a scale was constructed to measure agency in the healthcare context. The scale comprised five items assessing whether respondents took an active role in their interactions with the medical provider:

How often have you:

- Refused treatment offered or not followed advice or guidance given by your doctor/local surgery?
- Requested a particular treatment or test from a doctor/local surgery?
- Told your doctor/local surgery that you don’t agree with his/her opinion?
- Disagreed with your doctor/local surgery but did not say anything?
- Sought other treatment after a disagreement with your doctor/local surgery?

The response options ranged from 0 = never to 4 = always. A summary score was calculated ranging from 0 to 20. Internal consistency was acceptable (α = 0.71). Further analyses of the psychometric properties of the scale showed that it was closely related to the trust in physicians scale (r = − 0.51, *p* < 0.001). However, an explorative factor analysis including items from the health literacy, trust and agency scales showed that the items were loading on three distinct factors. For the analysis the score was grouped into tertiles based on the distribution in our sample (low = 0–2; medium = 3–6; high = 7–20).

### Analysis

Samples were weighted according to the age and gender distribution of the underlying population. Respondents were excluded from the analysis if they did not have a health concern since moving to the neighbourhood. Frequencies were used to describe sample characteristics. Prevalence of the types of bricolage was calculated as proportions with 95% confidence intervals (CI). As the outcome variable has several categories, determinants of bricolage were assessed by multinomial logistic regressions. Coefficients from multinomial regressions are difficult to interpret because they are relative to a base outcome. Therefore, we derived marginal effects from the multinomial regression using the postestimation *margins* command in Stata. This command estimates the effect of changing the values of the determinants on the probability of observing an outcome. Delta method was used for calculating 95% CIs. All analyses were carried out using Stata 12 (Stata Corp, College Station, Texas, US).

## Results

From the 2692 survey participants, 886 did not report any health concern and another 35 did not have valid responses for the outcome variable, leaving *n* = 1771 for analysis. The sample characteristics are shown in Table 1.

**Table 1:** Sample characteristics

Variables	n	%
Site, Country		
Birmingham, UK	333	18.8
Bremen, Germany	715	40.4
Lisbon, Portugal	261	14.7
Uppsala, Sweden	462	26.1
Age groups		
18–29 years	393	22.3
30–44 years	388	22.0
45–59 years	417	23.6
60–79 years	490	27.8
80 years or older	76	4.3
Gender		
Women	943	53.3
Men	825	46.6
Other	2	0.1
Education		
Low (ISCED 0–2)	506	29.2
Medium (ISCED 3–4)	611	35.3
High (ISCED 5–6)	616	35.6
Unemployed		
No	1613	91.1
Yes	158	8.9
Migration background		
None	1010	57.2
First generation	444	25.1
Second generation	221	12.5
Third generation	91	5.2
Language proficiency^a^		
Good	292	73.7
Poor	104	26.3
Years living in the country^a^		
0–10 years	98	26.3
11–20 years	97	26.0
> 20 years	178	47.8
Self-rated health		
Good	1242	70.2
Poor	526	29.8
Functional limitations		
Severely limited	550	31.2
Limited but not severely	745	42.3
Not limited at all	468	26.6
Trust in physicians^b^		
Low	379	30.6
Medium	535	43.2
High	325	26.3
Low health literacy^b^		
No	1141	86.8
Yes	173	13.2
Agency		
Medium	731	42.0
High	589	33.9
Medium	419	24.1

^a^assessed only among migrants

^b^not assessed in Sweden

Prevalence of using only the public healthcare system (PHS) to address the health concern (no bricolage) was 14.4% (95% CI: 12.7—16.3). Within-system bricolage was the most prevalent type in our sample (44.1%, 41.4—46.9), followed by added-to-system bricolage (33.5%, 30.8—36.2). Some 6.9% (5.6—8.4) used other resources and not the public system and 1% used no resources whatsoever. Since the latter group was very small, it was omitted in subsequent analyses. The distribution of the resources used within the types of bricolage can be found in Additional file 3: Table S3. The options offered in the questionnaire were identified during the qualitative phase and included public healthcare system, out-of-pocket services, alternative medicine, transnational practices, support from friends and family, internet and other informational sources.

**Fig. 1:**
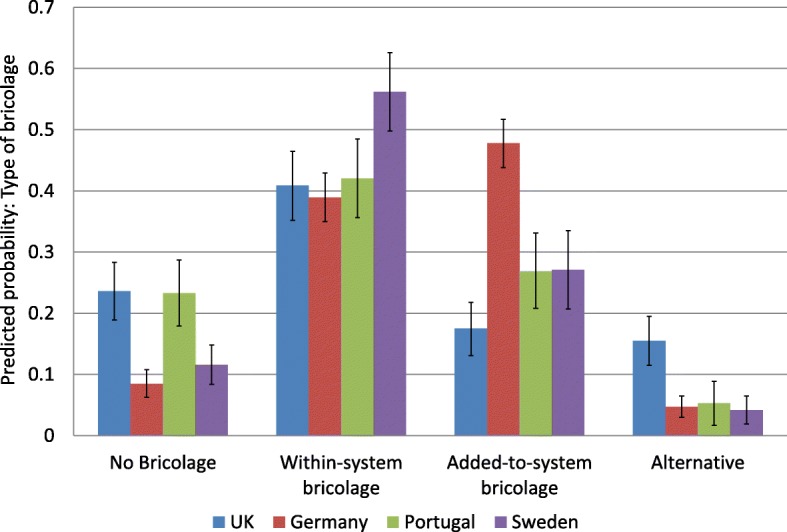
Frequency of bricolage tactics across the case study sites

Figure 1 shows the predicted probabilities for the types of bricolage across the four countries adjusted for age and gender. The probability of not engaging in bricolage was much higher in the UK and in Portugal compared to Germany and Sweden. Engaging in within-system bricolage was highest in Sweden and at comparable levels in the other three countries. Probability for adding to the system was particularly high in Germany and low in the UK. By contrast, avoiding the public health system and using alternatives was more common in UK than elsewhere.

The diversification of diversity that is a key characterise of superdiversity, implies the need to focus on the intersection between multiple variables including migration status, gender, age and education levels [1]. Associated with such diversification we have discussed elsewhere the importance of identifying the difference, or combination of differences which makes a difference to people’s access to healthcare [50]. Thus, it is important to understand which sociodemographic determinants shape bricolage behaviours rather than focus specifically upon ethno-national characteristics. The association between type of bricolage and sociodemographic determinants as well as health status are presented in Table 2. Being older increased the probability for no bricolage and reduced the probability of adding to the system. The survey data also showed that men were more likely not to bricolage while women were more likely to add to the system and that individuals with medium or higher educational degrees had increased probabilities of using additional resources or bypassing the public healthcare system. Survey findings around women engaging in higher levels of within system bricolage were unsurprising given the predominance of women in our semi-structured interviews reporting the work they undertook attempting to make public health systems work for their families. In Bradby et al. 2019 [51] we describe the significant practical, administrative and emotional efforts that women make in order to access appropriate care for their parent or offspring. The survey findings indicate that unemployed persons tended to do less adding to the system. This association was however not statistically significant. Survey findings showed that individuals with poor self-rated health were more likely to address a health concern by adding resources to the public healthcare system. Also, individuals with a health concern not resulting in functional limitations were less likely to use additional resources but more likely to bypass the system altogether.

**Table 2:** Sociodemographic determinants of bricolage (marginal effects, change in probability)

	No bricolage Coef. (95% CI)	Within-system Coef. (95% CI)	Added to system Coef. (95% CI)	Alternative Coef. (95% CI)
Age	.002 (.001; .003)*	.001 (.000; .003)	−.002(.004; .000)*	−.001 (−.002; .000)
Gender (ref. Women)				
Men	.06 (0.02, 0.09)*	.04 (−.01; .10)	−.11 (−.16; .06)*	.00 (−.02; .03)
Education (ref. Low)				
Medium	−.10 (−.16; −.05)*	.02 (−.06; .11)	.00 (−.08; .09)	.07* (.04; .11)
High	−.11 (−.17; −.06)*	−.03 (−.12; .05)	.09 (.00; 0.18)*	.04* (.01; .08)
Unemployed (ref. No)				
Yes	.01 (−.05; .07)	.03 (−.07; .13)	−.05 (−.15; .02)	−.00 (−.05; .06)
Self-rated health (ref. Good)				
Poor	.00 (−.04; .04)	−.06 (−.13; .00)	.09 (.02; .17)*	−.02 (−.06; .01)
Functional limitations (ref. Severely limited)				
Limited but not severely	−.01 (−.05; .03)	.03 (−.04; .09)	−.05 (−.11; .01)	.03 (.00; .06)
Not limited at all	.04 (−.01; .09)	−.01 (−.08; .06)	−.08 (−.14; −.01)*	.04 (.01; .08)*

Adjusted for survey country

*p < .05

Table 3 shows the association of migration-related variables with types of bricolage. Overall, individuals with a migration background tended to be less likely than those without a migration background to engage in within-system bricolage. This may be because they lack connections with individuals capable of helping them to navigate the system [52]. This difference was greatest for second generation migrants (− 11 percentage points). Other than reduced tendency to within-system bricolage, there were no clear trends relating to length of residence in the survey data. The survey data found no relationship between language proficiency and patterns of bricolage.

**Table 3:** Migration-related determinants of bricolage (marginal effects, change in probability)

	No bricolage Coef. (95% CI)	Within-system Coef. (95% CI)	Added to system Coef. (95% CI)	Alternative Coef. (95% CI)
Migration background (ref. None)				
1st Generation	.03 (−.02; .07)	−.06 (−.13; .01)	.04 (−.03; .11)	−.01 (−.05; .02)
2nd Generation	.01 (−.05; .08)	−.11 (−.20; −.01)*	.06 (−.04; .16)	.00 (−.04; .04)
3rd Generation	−.04 (−.12; .04)	−.07 (−.20; .05)	.08 (−.03; .19)	.03 (−.04; .10)
Years living in the country (ref. Not migrant)				
0–10 years	.00 (−.08; .09)	−.10 (−.23; .02)	.06 (−.07; .19)	.03 (−.02; .08)
11–20 years	.06 (−.03; .15)	.03 (−.11; .17)	−.03 (−.17; .11)	−.06 (−.09; −.04)*
> 20 years	.04 (−.01; .09)	.02 (−.06; .11)	−.04 (−.12; .05)	−.01 (−.06; .03)
Language proficiency (ref. Not migrant)				
Good	.03 (−.02; .08)	−.04 (−.12; .04)	.02 (−.07; .19)	−.01 (−.04; .02)
Poor	.02 (−.05; .08)	.01 (−.10; .12)	.00 (−.11; .11)	−.02 (−.07; −.02)

Adjusted for survey country, age, gender, education, and functional limitations

*p < .05

### Health literacy

As we discuss above, levels of health literacy are frequently used as a measure of patients’ ability to comprehend and act upon medical instructions and have been associated with poor outcomes for minority and migrant groups. In the unadjusted analysis, there was a trend that those with a low health literacy were less likely to engage in bricolage (Table 4). Specifically, they tended to do less adding to the system or seeking help outside the system. However, these associations were not statistically significant and became even weaker after controlling for age, education, migration background (all correlated with health literacy) and other variables. In the semi-structured interviews, we found stronger evidence that low levels of health literacy obstructed access to the public healthcare system and sometimes led to individuals engaging in within system bricolage aided by others to use public healthcare. Several respondents described struggling to understand how to use the healthcare system or to know if they were entitled to use the system. Some had no information about the range of services on offer, which meant they did not attempt to use services. For example, in Germany Ols (34, economic migrant originating in Gambia) was unaware that she could access midwives to help monitor her pregnancy so had no contact with antenatal care.

**Table 4:** Health-related knowledge, attitudes and experiences as determinants of bricolage (marginal effects, change in probability)

	No bricolage Coef. (95% CI)	Within-system Coef. (95% CI)	Added to system Coef. (95% CI)	Alternative Coef. (95% CI)
Trust in physicians				
Unadjusted (ref. Low)				
Medium	.07 (.02; .12)*	.06 (−.01; .13)	−.18 (−.24; −.11)*	.04 (.00; .08)*
High	.11 (.05; .16)*	.09 (.01; .16)*	−.25 (−.32; .17)*	.05 (.01; .10)*
Adjusted^a^ (ref. Low)				
Medium	.03 (−.01; .07)	.06 (−.01; .13)	−.10 (−.16; −.03)*	−.01 (−.02; .05)
High	.01 (−.03; .06)	.07 (−.01; .16)	−.11 (−.18; .02)*	−.02 (−.02; .07)
Agency (ref. Low)				
Unadjusted (ref. Low)				
Medium	−.13 (−.17; −.09)*	.03 (−.03; .09)	.15 (.09; −.20)*	−.03 (−.06; .01)
High	−.18 (−.22;. -14)*	−.13 (−.20; −.06)*	.35 (.28; .42)*	−.02 (−.06; .02)
Adjusted^a^ (ref. Low)				
Medium	−.07 (−.11; −.02)*	.01 (−.05; .08)	.09 (.03; −.15)*	−.02 (−.05; .01)
High	−.12 (−.16;. -08)*	−.11 (−.19; −.04)*	.25 (.17; .33)*	−.01 (−.05; .03)
Health literacy				
Unadjusted (ref. High)				
Low	.06 (.00; .13)	.02 (−.06; .11)	−.05 (−.11; .04)	−.04 (−.08; .01)
Adjusted^a^ (ref. High)				
Low	.00 (−.04; .04)	.02 (−.05; .08)	−.02 (−.08; .04)	.00 (−.04; .05)

^a^Adjusted for survey country, age, gender, education, migration background and functional limitations

*p < .05

Within system bricolage was frequently facilitated by friends and family, NGOs and the internet for those reporting low levels of health literacy. Individuals with small or no networks and no access to online information struggled to understand how to use services. The movement of much information about public healthcare online was reported to be problematic for some respondents – they needed awareness of search terms and access to appropriate equipment and Wi-Fi. While migrants, many of whom were under the age of 45, appeared more likely to have low levels of health literacy compared with other relatively young people, lack of knowledge about how to use the system was not a specifically migrant concern. Elderly people and individuals with addiction problems also reported struggling to understand which services were available. Individuals with low levels of health literacy were sometimes helped by NGOs to access public healthcare systems. The Santa Casa da Misericordia, lottery-funded parallel healthcare system and Doctors of the World in Lisbon were particularly active in reaching out to substance abusers, the homeless and isolated elderly people and brokering access. Some respondents were not able to find help to understand treatment options. For example, Eurico, a 38-year-old Portuguese man and full-time career of his five-year-old son who had a very rare condition, explained he knew little about his son’s condition and found the explanations given by health providers unintelligible. They waited over 3 years for an important operation but lacked the knowledge or the resources to find out whether they could expedite the operation or access alternative treatments.

### Trust

The survey data indicated that trust in physicians was strongly related to bricolage (Table 4) supporting findings from residents’ semi-structured interviews. Survey results showed that high levels of trust tended to increase the probability of no bricolage and within-system bricolage and reduce the probability for adding to the system by 25 percentage points. The relationship between lack of trust in the services provided by the public healthcare system and adding to the system was evident in interviews in all four cities. Not being able to access a familiar healthcare professional who knew your medical history and whom you felt had some kind of vested interest in your care made individuals feel they were “just a number” and was said to undermine trust. Respondents’ feelings that they could not trust the system were reported to relate to not getting an optimal service or believing they were misdiagnosed or mistreated. Interviewees who reported low levels of trust said this feeling led them to seek other kinds of resources: to pay for a second opinion (although in Germany this was routine), to check on their diagnosis, seek a different specialist or treatment or to opt out of the system altogether.

Feeling that they were being discriminated against appeared to lower levels of trust in the system and resulted in people adding additional services. For example, a Chilean-born 24-year old student in Uppsala described how his mistrust resulted from attending Emergency services with his mother and seeing ethnic Swedes access services more quickly than them. He reported they waited for ‘*about two hours although there weren’t many people waiting to be seen*.’ He went on to say his mother only received help after she fainted at which point she got to ‘*lie in a bed, was given various blood tests and other tests*’. Following these experiences, the entire family was said to actively avoid the system, preferring to self-treat or pay for private services.

In Germany older respondents highlighted the loss of services following centralization as undermining their trust in local public health services. In all the countries some respondents reported strong and enduring relationships with specialists who helped them to deal with long-term conditions but also assisted with unrelated health concerns when asked for help. This we consider a form of within system bricolage. For example, Ludwig, aged 64, of German origin only felt confident to seek a medical consultation when he had a trusting relationship with a clinician. Given that he did not know any of the doctors at his local surgery, he asked his specialist about unrelated medical problems and followed this advice rather than seek out the local doctors who had responsibility for Ludwig’s general health.

Individuals who did no bricolage said they remained within the public health system because they trusted it or at least trusted their own doctor. The experience of respondents in the UK was notable with several reporting knowing their doctor for years and occasionally describing them as “*a family friend*”. Others talked about having good experiences which meant they did not need to seek alternatives. For example, in Portugal Elsa, 60, from Colombia, reported positive experiences at her local health centre so said she felt no need to seek help elsewhere. Interviews in Sweden identified more individuals who stated that they were very satisfied with the system and felt that all their needs were met than in the other case study countries. As a 69 year-old Swedish woman who had retired through ill health, said when asked whether she trusted in the healthcare system stated “*I’ve had no reason NOT to trust it … ..It has worked for me. It has met my needs*”.

### Agency

The survey findings showed that levels of agency were also strongly related to the tendency to bricolage (Table 4). In the adjusted model, a medium or high level of agency reduced the probability of not engaging in bricolage by 7 and 12 percentage points, respectively. Interviews showed that agency was important to individuals engaging in both within-system and adding to the system bricolage. For instance, Isabella, a 33-year-old Brazilian born woman, had experienced stress-related alopecia for several months. Having received no treatment from the general practitioner she began a quest to address her hair loss. She utilized multiple resources include paying to see private specialists in Portugal, France and Brazil, engaging in yoga and Chinese treatments, importing vitamin supplements and finally some medication from Brazil. Her actions demonstrated her preparedness to engage in multiple endeavours to reach a satisfactory outcome, even if a cure was not possible. In both Portugal and Sweden individuals reported deciding to use emergency services because they knew they would access a specialist more quickly than waiting for a referral from primary care. Survey findings showed that a high level of agency increased the probability of adding-to-system bricolage by 25 percentage points after controlling for age, gender, migration background and other variables.

While we may argue that adding resources to the formal system usually engenders some kind of agency, there were individuals who were clear that information seeking was something they needed to do to retain control over treatment of their health concern. Information enabled them to communicate effectively with their doctor and know whether they were getting the correct treatment suggesting that for them bricolage could be viewed in itself as a form of agency. For example, Anna, 31, an artist originating in Germany recounted spending much time studying medical books so that she could retain control of her treatments by becoming “*an expert*”. Jay, a 40-year-old Project Manager of Ghanaian origin visited the general practitioner in Germany on three occasions and was diagnosed with a back condition and depression. However, he refused a back operation, instead speaking to a specialist in Ghana and seeking out a chiropractor. He also declined counselling and chose instead to confide in his mother and to pray. He used the public healthcare system for diagnoses but wanted to feel in control of his care and to avoid the possibility of side effects. Several other respondents reported seeking diagnoses and then making their own decisions about whether to accept treatment. Several refused surgeries preferring to treat their conditions in a less invasive way. Elsewhere some respondents rejected the medication offered by their medical professional and others took biomedicine sent from abroad because they were more familiar with that treatment and used familiarity to retain control. Respondents with low levels of agency sometimes felt they had no choice but to use the formal system. This was often because they were not able to access knowledge necessary to seek out alternatives. For instance, a Swedish 64-year-old teacher, of Chilean origin explained that ‘*he was not knowledgeable in medical questions*’ and therefore he simply trusted doctors. ‘*What should I do? Should I sit and discuss with them?*’ he said, laughing.

## Discussion

Our findings demonstrate that several factors shaped individuals’ tendency to bricolage and the types of bricolage in which they engaged. The nature, duration and severity of the health concern influenced actions, for instance fractured bones necessitated attendance at emergency clinics whereas chronic conditions such as alopecia that did not limit function lent themselves to multiple actions over an extended period of time. Severity and limitation of function were important factors that prompted individuals to engage in bricolage. Semi-structured interview data showed that lack of relief or resolution of long-term or chronic conditions were important factors that prompted respondents to search for additional resources thereby engaging in adding to the system bricolage. The tendency to, and type of, bricolage were also related to levels of education, age, gender, and migrant background suggesting that a focus on superdiversity, and the differences that make a difference are important. Semi-structured interview data showed that ability to access internet-based knowledge and even consultations, and to understand and navigate complex public health systems, were contingent on age and education level. While survey data did not show a significant association between employment status and type of bricolage, the semi-structured interviews supported the direction of the association found in the survey data. Thus, in relation to adding to the system bricolage respondents with higher incomes could afford out of pocket services while those on low incomes would have liked to have used such services but had to use low or no cost approaches. Bricolage is evidently gendered with men less likely to bricolage and women more likely to engage in added to system bricolage. The reasons for this are unclear but may be connected to women’s greater likelihood of reporting symptoms and of using the public healthcare system compared with men [53], suggesting that bricolage should be understood as an extension of wider processes of gendered healthcare access [49].

Crucially agency and trust come through in the survey and interviews as particularly important in determining bricolage tactics. Such findings are important because interventions which increase trust and agency offer potential to encourage people to seek help when needed [30]. We find that agentic behaviour underpins within-system, adding to the system and alternative to the system, bricolage, while individuals with low levels of agency do not make treatment demands of clinicians sometimes resulting in them attending repeated clinical consultations without resolution. Agency helped some respondents to access treatments more quickly through making “appropriate” demands of clinicians rather than waiting to be offered treatment. While health literacy did not emerge as important in our survey, the qualitative findings show that cultural health capital [54], knowing how to act in a consultation, was important in terms of within-system bricolage. Respondents who reported lacking such capital could still benefit from it if someone in their social network could help them maneuver through the system, reflecting earlier work on the importance of navigation [52]. The contradiction between our survey and interview findings suggest we need to rethink our research instruments since agency, literacy and trust are not only individual qualities, but are also properties of networks which may be local, transnational, formal and informal.

The aim of the UPWEB project was to examine the ways in which people enacted multiple resources to address healthcare concerns. The heuristic of healthcare bricolage enables us to focus on actions as embedded into local contexts, the resources individuals employ and the reasons they employ them. Bricolage moves beyond the notion of pluralism by starting from the perspective of healthcare users, to understand their actions and priorities, rather than privileging professional perspectives and interactions between systems. Furthermore, bricolage allows a range of different scales to be considered simultaneously - local, digital, national, transnational – whereas pluralism has tended to focus on the interaction of two systems within one society. Through examining bricolage, we can learn much about how people act, in what circumstance and with which resources. Our focus has been on superdiverse neighbourhoods because we were interested in “additional” resources such as culturally specific treatments, wide ranging knowledges and multitudinous transnational connections that might bring opportunities to areas that are often seen as problematic in terms of health access and outcomes. It would be useful to explore how the concept can be utilized in other environments.

For instance, the extent to which bricolage solutions are adopted in community-based care is probably greater than might be ascertained from standard audit data, since informal solutions tend not to be reported through official channels. Making visible work that would otherwise remain outwith the analysis of healthcare work, and the extent to which this work is via networks, rather than individual capabilities are important findings. Further research is required to explore whether certain patterns or processes are beneficial and have potential to be connected to existing public healthcare system, or to be presented as alternatives in relation to certain types of concern, to improve outcomes and levels of patient satisfaction and reduce pressure in overburdened systems.

## Conclusions

The notion of healthcare bricolage has the potential to offer a heuristic to highlight actions that may be viewed as beneficial or detrimental to health in policy terms and to offer insights into practices that have the potential to bring innovation into rather inflexible public healthcare systems. Examining the reasons why people engage in bricolage can also help us to learn about the aspects of systems that are problematic for users.

As a consequence, we may be able to address problematic areas for example by encouraging the development of trust through ensuring continuity of care or ensuring that trainee clinicians are educated about the importance of understanding that there are multiple cultural health capitals [54]. We did not seek to identify if, and when, bricolage is effective in addressing individuals’ health concerns or the relative effectiveness of some kinds of bricolage over others. We examine in a separate paper the bricolage tactics adopted by healthcare providers in order to reduce the pressure of demand in superdiverse neighbourhoods [12]. Having demonstrated the presence of variable tactics of healthcare bricolage in both patients and healthcare providers, we argue that the concept can be further utilized to highlight that the functioning of biomedical systems frequently depends on actors’ usage of multiple unacknowledged social, economic and cultural resources.

## Supplementary information

**Additional file 1:** Interview guide.

**Additional file 2:** Questionnaire.

**Additional file 3:** **Table S1.** Characteristics of the comparison countries and neighbourhoods. **Table S2.** Qualitative Interviewee profiles. **Table S3.** Resources used within the types of bricolage.

## Data Availability

Raw data is not available as consent was not received for data sharing. Thus datasets generated and/or analysed during the current study are not publicly available for reasons of confidentiality (the grant did not include costs of anonymisation) but by negotiation pooled datasets may be made available on request via the lead author. Research tools have been uploaded as Additional Materials.

## Abbreviations

CI: Confidence intervals
HLSEU-Q6: European Health Literacy Survey Questionnaire (6-item Version)
ISCED: International Standard Classification of Education
NHS: National Health Service
PHS: Public healthcare system
UK: United Kingdom
UPWEB: Understanding the practice and developing the concept of welfare bricolage
