# Supplementary material for: Healthcare bricolage in Europe’s superdiverse neighbourhoods: a mixed methods study
Source: BMC Public Health. 2019 Oct 22;19:1325. doi: 10.1186/s12889-019-7709-x (PMC6805362; doi:10.1186/s12889-019-7709-x)
Supplement: Supplementary file 1 — Additional file 1: Interview guide. [file 12889_2019_7709_MOESM1_ESM.docx]

# UPWEB INTERVIEW QUESTIONS

**OPENING QUESTIONS – STAYING HEALTHY**

1. What kinds of things do you do to stay healthy?
2. What matters to you most in relation to your health? What do you think affects your health most?
3. What additional activities would you like to do or what support you need to help you to be healthy?
4. What do you normally do if you have a health problem?

**TIMELINE QUESTIONS**

1. Since you were living in this area when were you last ill or had a health worry and felt you needed to get some help? (use timeline)

Probes: What did you do? How long did you wait? Interested in everything, who did you ask, who suggested that, did you use internet, what for, who helped/ supported – in what way, how did they make you feel, what were the gaps, what did not go well, what didn’t you do that you wanted to do, what might have been done differently (by you or one of the health providers) (use a checklist to make sure you have covered all of the possible sources of help (formal, informal, complementary, alternative, faith, internet, skype…), blockages or divergences

1. What is the most serious health problem you have experienced? (use timeline)

Probes: What did you do? How long did you wait? Interested in everything, who did you ask, who suggested that, did you use internet, what for, who helped/ supported – in what way, how did they make you feel, what were the gaps, what did not go well, what didn’t you do that you wanted to do, what might have been done differently (by you or one of the health providers) (use a checklist to make sure you have covered all of the possible sources of help (formal, informal, complementary, alternative, faith, internet, skype…), blockages or divergences If no serious problem – ask about a person living in the same house as you or maternity?

ADDITION TO Q5 and Q6 (if those answers are short)

1. If the incidents recalled do not reveal much and you have time you can ask Have you experienced any other health problems that you have needed help with?

**BROADER HEALTH ISSUES**

1. What are the biggest issues around health and wellbeing for your friends, and people within your community?
2. How do people around you and in your community get help with mental-health related issues? (Adapt language for specific cultural context)
3. Who do you trust most in relation to your healthcare?
4. In terms of the help you have received with health problems what do you think has been the most useful to you? Why?
5. What has been the biggest barrier to what you are able to do in order to address your health problems?

**CONCLUDE.**

**Notes**

Timeline – mark all the events on the timeline with the interviewee and annotate nodes saying what happened at that moment – make sure you verbalise what you are doing so that the transcripts can stand alone

We will produce a checklist setting out all of the possible sources of help/support in our topic guide – after the open/narrative question we check to see if they used any of these sources

Remember to ask for suggestions of further interviewees at the conclusion of the interview.
