# Supplementary material for: Healthcare bricolage in Europe’s superdiverse neighbourhoods: a mixed methods study
Source: BMC Public Health. 2019 Oct 22;19:1325. doi: 10.1186/s12889-019-7709-x (PMC6805362; doi:10.1186/s12889-019-7709-x)
Supplement: Supplementary file 2 — Additional file 2: Questionnaire. [file 12889_2019_7709_MOESM2_ESM.docx]

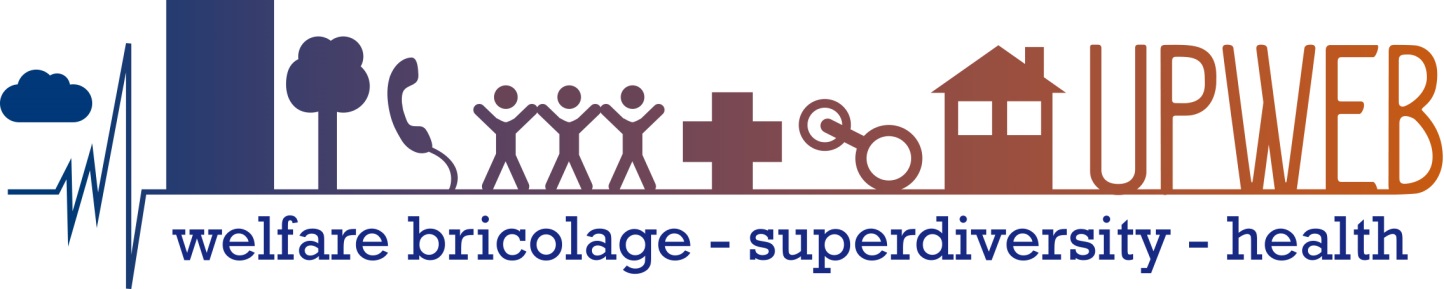


UPWEB – Understanding the practice and developing the concept of welfare bricolage

English Version

| **Variable name** |  | |  |
| --- | --- | --- | --- |
| PID | Respondent’s ID | __\|__\|__\|__\|__\|__\| |  |
| Int1 | Interviewer ID | __\|__\|__ |  |
| Int2 | Neighbourhood | __ _1_ Edgbaston  __ _2_ Handsworth  __ _3_ Neustadt  __ _4_ Gröpelingen  __ _5_ Mouraria  __ _6_ Lumiar  __ _7_ Gottsunda  __ _8_ Sävja |  |
| Int3 | Interview language | __ _1_ German  __ _2_ English  __ _3_ French  __ _4_ Turkish  __ _5_ Arabic  __ _6_ Bulgarian |  |
| Int4 | Date | __\|__. __\|__. __\|__\|__\|__  TT MM JJJJ |  |
| Int5 | Time (Start) | __\|__. __\|__  Std. Min. |  |

**Thank you once again for agreeing to participate in this interview. During the next half an hour I will ask you a few questions concerning your health, your access to health services and your living conditions.**

**If you don’t have any questions, I will start with the first questions.**

| **General Information** |
| --- |

**To begin with I’d like to ask you some general questions.**

**Age**

**Q1 How old are you?**

__|__ Years __ _998_ [Don’t know] __ _999_ [Refused]

**Gender**

**Q2 What is your gender?**

__ _1_ Male

__ _2_ Female

__ _3_Other

__ _8_ [Don’t know]

__ _9_ [Refused]

**Self-rated health**

**Q3 In general, would you say that your health is**

*INT: Present show card 1*

__ _1_ Excellent

__ _2_ Very good

__ _3_ Good

__ _4_ Fair

__ _5_ Poor

__ _8_ [Don’t know]

__ _9_ [Refused]

**Health insurance status**

**Q4** **Are you currently covered by a health insurance?**

*INT: Present show card 2*

__ _1_ Yes, statutory health insurance

__ _2_ Yes, private health insurance

__ _3_ Yes, other 🡪 [Q4a] Which one?

_________________________________________________________________________

__ _4_ No

__ _8_ [Don’t know]

__ _9_ [Refused]

| Bricolage |
| --- |

**Now we’ll focus on health care here in the neighbourhood.**

**Q5 In which year did you come to live in this neighbourhood?**

__|__|__|__ *INT: Write in year*

**Q5a** **Since you moved to this neighbourhood, have you had a health concern? By health concern, I mean a physical or mental condition or disease.**

*INT: This also includes concerns that have been resolved or started before the respondent moved to the neighbourhood. Present show card 3 if the respondent is unsure or says no.*

__ _1_ Yes

__ _2_ No

* If No or d.k/ref. please continue with* ***Q17***

__ _8_ [Don’t know; d.k.]

__ _9_ [Refused; ref. ]

**Q6a** **Which health concern did you have?**

*INT: If more than one then ask for the one they felt was more/most important to them. Present show card 3 if the respondent is unsure.*

_____________________________________________________________________

**Q6b To what extent were you limited in your normal everyday activities, when you had this health concern?**

*INT: Present show card 4*

__ _1_ Severely limited

__ _2_ Limited but not severely

__ _3_ Not limited at all

__ _8_ [Don’t know]

__ _9_ [Refused]

**Q7a Thinking about this health concern, did you use services provided by the public healthcare system to address it?**

*INT: Present show card 5*

*If Yes or d.k./ref., please continue with* ***Q8a***

__ _1_ Yes

__ _2_No

__ _8_ [Don’t know]

__ _9_ [Refused]

**Q7b What was the main reason for not using the public healthcare system? Here are some answers that are commonly given:**

*INT: Present show card 6*

| __ _1_ | Did not need to |
| --- | --- |
| __ _2_ | Not entitled to use local services |
| __ _3_ | Did not have time because of work, care for children or for others |
| __ _4_ | Waiting list was too long |
| __ _5_ | Too far to travel / no means of transportation |
| __ _6_ | Didn’t know which services might be suitable |
| __ _7_ | Services available deemed not to be acceptable – for example, quality of services, culturally inappropriate, against one’s beliefs |
| __ _8_ | Communication with service providers difficult |
| __ _9_ | Preferred to treat myself |
| __ _10_ | Other reason 🡪[Q7c] What other reason?  _______________________________________________________________ _______________________________________________________________ |
| __ _98_ | [Don’t know] |
| __ _99_ | [Refused] |

**Q8a Did you use healthcare services that you had to pay for completely out of your own pocket to address the concern?**

*INT: Present show card 7*

__ _1_ Yes

__ _2_ No

*If no or d.k./ref., please continue with* ***Q9a***

__ _8_ [Don’t know]

__ _9_ [Refused]

**Q8b What was the main reason for using out-of-pocket services? Was it because…**

*INT: Present show card 8*

| __ _1_ | …you wanted to get a second opinion/ a further diagnosis |
| --- | --- |
| __ _2_ | …you wanted to avoid a long waiting list for standard service |
| __ _3_ | …you wanted some treatment whilst on waiting list for state provided services |
| __ _4_ | … state provided services received did not address your concern |
| __ _5_ | …services required were not provide by the state |
| __ _6_ | …you felt that the treatment would be good for you in addition to the state provided services |
| __ _7_ | Other reason 🡪 [Q8c] What other reason? _______________________________________________________________ _______________________________________________________________ |
| __ _8_ | [Don’t know] |
| __ _9_ | [Refused] |

**Q9a Did you use alternative or complementary medicine to address the concern?**

*INT: Present show card 9*

__ _1_ Yes

__ _2_ No

*If no or d.k/ref. please continue with* ***Q10a***

__ _8_ [Don’t know]

__ _9_ [Refused]

**{Q9b-e}** **What kind of alternative or complementary medicine did you use?**

*INT: Still show card 9, multiple answers possible.*

__ _1_ [Q9b] Natural products

__ _1_ [Q9c] Body and mind practices

__ _1_ [Q9d] Spiritual healing

__ _1_ [Q9e] Other health practices

__ _8_ [Don’t know]

__ _9_ [Refused]

**Q9f What was the main reason for using alternative or complementary medicine? Here are some answers that are commonly given:**

*INT: Present show card 10*

| __ _1_ | To get a second opinion/ a further diagnosis | |
| --- | --- | --- |
| __ _2_ | To avoid a long waiting list for standard service | |
| __ _3_ | Wanted some treatment whilst on waiting list for state provided services | |
| __ _4_ | State provided services received did not address your concern | |
| __ _5_ | Always use alternative or complementary medicine | |
| __ _6_ | Other reason [Q9g] What other reason? 🡪  _______________________________________________________________ _______________________________________________________________ |  |
| __ _8_ | [Don’t know] | |
| __ _9_ | [Refused] | |

**Q10a Did you use treatment, medicine or medical advice from another country to address the concern?**

*INT: Present show card 11*

__ _1_ Yes

*If no or d.k./ref. please continue with* ***Q11***

__ _2_ No

__ _8_ [Don’t know]

__ _9_ [Refused

**Q10b What was the main reason for using treatment, medicine or medical advice from another country? Here are some answers that are commonly given:**

*INT: Present show card 12*

| __ _1_ | To get a second opinion/ a further diagnosis |
| --- | --- |
| __ _2_ | To avoid a long waiting list for standard service |
| __ _3_ | Wanted some treatment whilst on waiting list for state provided services |
| __ _4_ | Service received locally did not address your concern |
| __ _5_ | Unable to find the services/ treatments/ medications you wanted locally |
| __ _6_ | Services offered in the other country were cheaper |
| __ _7_ | Preferred to use familiar/trusted treatment/medicine |
| __ _8_ | Was not entitled to use local health services |
| __ _9_ | Other reason 🡪 [Q10c] What other reason? _______________________________________________________________ _______________________________________________________________ |
| __ _98_ | [Don’t know] |
| __ _99_ | [Refused] |

**Q11 Did you seek advice or support from family, friends etc. to address the concern?**

*INT: Present show card 13*

__ _1_ Yes

__ _2_ No

__ _8_ [Don’t know]

__ _9_ [Refused]

**Q12 Did you use information from the internet?**

__ _1_ Yes

__ _2_ No

__ _8_ [Don’t know]

__ _9_ [Refused]

**Q13 Did you use information from other sources?**

*PROBE: By other sources I mean books, magazines, radio, TV etc.*

__ _1_ Yes

__ _2_ No

__ _8_ [Don’t know]

__ _9_ [Refused]

**Q14** INT: *If more than one resource was used:*

**In the previous set of questions we asked you about the different health services / treatments that you used regarding your health concern. Which of these resources did you use first?**

*INT: Show card 14*

__ _0_ [Not more than one resource used]

__ _1_ Services provided by the public healthcare system

__ _2_ Medical care paid completely out of pocket

__ _3_ Alternative or complementary medicine or treatment

__ _4_ Treatment, medicine or medical advice from another country

__ _5_ Help or advice from family, friends etc.

__ _6_ Information from the internet

__ _7_ Information sources excluding internet

__ _8_ [Don’t know]

__ _9_ [Refused]

**Q15 On a scale from 1 (never) to 5 (always), how often in the last five years have you used more than one approach to deal with a health concern?**

*INT: Present show card 15*

*PROBE: By more than one approach I mean the combination of services or activities, such as looking for information in the internet, using the public healthcare system, seeking private healthcare etc.*

__ _1_ Never

__ _2_ Hardly ever

__ _3_ Sometimes

__ _4_ Often

__ _5_ Always

__ _8_ [Don’t know]

__ _9_ [Refused]

**Q16 How often did you visit your family doctor/general physician in the past 12 months?**

*INT: Present show card 16*

__ _1_ Never

__ _2_ 1 to 2 times

__ _3_ 3 to 5 times

__ _4_ 6 to 10 times

__ _5_ More than 10 times

__ _8_ [Don’t know]

__ _9_ [Refused]

*INT: Present show card 17*

**The next set of questions cover resources that you generally find most useful when addressing a health concern.**

**Which of these resources are most useful when you need the following:**

*PROBE: Please choose only one resource*

*PROBE: Instead of “most useful” use “do you rely on the most”*

**Q17a Find out what your health concern is**

| __ _1_ | Services provided by the public healthcare system/ NHS |
| --- | --- |
| __ _2_ | Services paid out of pocket |
| __ _3_ | Alternative or complementary medicine 🡪 Which one?  *INT: Multiple answers possible*  __ _1_ [Q17b]Natural Products  __ _1_ [Q17c]Body and mind practices  __ _1_ [Q17d] Spiritual Healing  __ _1_ [Q17e] Other health practices |
| __ _4_ | Services from another country |
| __ _5_ | Family, friends etc. |
| __ _6_ | Information from the internet |
| __ _7_ | Other information sources excluding the internet) |
| __ _8_ | [Don’t know] |
| __ _9_ | [Refused] |

**Q17f Information about prescription drugs**

| __ _1_ | Services provided by the public healthcare system/ NHS |
| --- | --- |
| __ _2_ | Services paid out of pocket |
| __ _3_ | Alternative or complementary medicine |
| __ _4_ | Services from another country |
| __ _5_ | Family, friends etc. |
| __ _6_ | Information from the internet |
| __ _7_ | Other information sources excluding the internet |
| __ _8_ | [Don’t know] |
| __ _9_ | [Refused] |

**Q17g Information about other possible treatments**

| __ _1_ | Services provided by the public healthcare system/ NHS |
| --- | --- |
| __ _2_ | Services paid out of pocket |
| __ _3_ | Alternative or complementary medicine |
| __ _4_ | Services from another country |
| __ _5_ | Family, friends etc. |
| __ _6_ | Information from the internet |
| __ _7_ | Other information sources excluding the internet |
| __ _8_ | [Don’t know] |
| __ _9_ | [Refused] |

**Q17h A recommendation for a specialist, hospital or other medical facility**

| __ _1_ | Services provided by the public healthcare system/ NHS |
| --- | --- |
| __ _2_ | Services paid out of pocket |
| __ _3_ | Alternative or complementary medicine |
| __ _4_ | Services from another country |
| __ _5_ | Family, friends etc. |
| __ _6_ | Information from the internet |
| __ _7_ | Other information sources excluding the internet |
| __ _8_ | [Don’t know] |
| __ _9_ | [Refused] |

**Q17i Emotional support in dealing with a health concern**

| __ _1_ | Services provided by the public healthcare system/ NHS | |
| --- | --- | --- |
| __ _2_ | Services paid out of pocket | |
| __ _3_ | Services from another country 🡪Which one?  *INT: Multiple answers possible*  __ _1_ [Q17j]Natural Products  __ _1_ [Q17k]Body and mind practices  __ _1_ [Q17l] Spiritual Healing  __ _1_ [Q17m] Other health practices | |
| __ _4_ | Services from another country |  |
| __ _5_ | Family, friends etc. |  |
| __ _6_ | Information from the internet |  |
| __ _7_ | Other information sources excluding the internet |  |
| __ _8_ | [Don’t know] |  |
| __ _9_ | [Refused] |  |

**Q17n Practical advice for coping with day-to-day situations e.g. pain, discomfort**

| __ _1_ | Services provided by the public healthcare system/ NHS |
| --- | --- |
| __ _2_ | Services paid out of pocket |
| __ _3_ | Alternative or complementary medicine |
| __ _4_ | Services from another country |
| __ _5_ | Family, friends etc. |
| __ _6_ | Information from the internet |
| __ _7_ | Other information sources excluding the internet |
| __ _8_ | [Don’t know] |
| __ _9_ | [Refused] |

| **Experiences at the doctor’s or health care centre** |
| --- |

**The next questions will now focus on your experiences at the doctor’s or health care centre.**

**How often have you:**

*INT: Present show card 18; instead of doctor’s also other healthcare professionals such as nurses, doctor’s assistants or midwives, are meant.*

**Q18a …refused treatment offered or not followed advice or guidance given by your doctor/health care provider?**

__ _1_ Never

__ _2_ Hardly ever

__ _3_ Sometimes

__ _4_ Often

__ _5_ Always

__ _8_ [Don’t know]

__ _9_ [Refused]

**Q18b** **…requested a particular treatment or test from a doctor/health care provider?**

__ _1_ Never

__ _2_ Hardly ever

__ _3_ Sometimes

__ _4_ Often

__ _5_ Always

__ _8_ [Don’t know]

___9_ [Refused]

**Q18c Told your doctor/health care provider that you don’t agree with his/her opinion?**

__ _1_ Never

__ _2_ Hardly ever

__ _3_ Sometimes

__ _4_ Often

__ _5_ Always

__ _8_ [Don’t know]

__ _9_ [Refused]

**Q18d Disagreed with your doctor/health care providerr but did not say anything?**

__ _1_ Never

__ _2_ Hardly ever

__ _3_ Sometimes

__ _4_ Often

__ _5_ Always

__ _8_ [Don’t know]

__ _9_ [Refused]

**Q18e Sought other treatment after a disagreement with your doctor/health care provider?**

__ _1_ Never

__ _2_ Hardly ever

__ _3_ Sometimes

__ _4_ Often

__ _5_ Always

__ _8_ [Don’t know]

__ _9_ [Refused]

**Discrimination and health**

**Q19 Would you describe yourself as someone who is discriminated against by health care providers in this country?**

__ _1_ Yes

__ _2_No

*If no or d.k./ref. please continue with* ***Q20***

__ _8_ [Don’t know]

__ _9_ [Refused]

**{Q19a-j} On what grounds are you discriminated against?**

*INT: Present show card 19*

*PROBE: What other grounds?*

__ _1_ Colour or Race

__ _1_ Nationality

__ _1_ Religion

__ _1_ Language

__ _1_ Ethnicity

__ _1_ Age

__ _1_ Gender

__ _1_ Sexuality

__ _1_ Disability

__ _1_ Other 🡪 [Q19g] Which one? ________________________________________

__ _8_ [Don’t know]

__ _9_ [Refused]

**Q20 Have you ever been refused treatment or consultation by a health professional since you’ve been living in this neighbourhood?**

*PROBE: By health professional I mean people working in the public healthcare sector such as GPs, specialists, nurses, midwives, doctor's receptionist.*

__ _1_ Yes

__ _2_ No

*If no or d.k./ref. please continue with* ***Q22***

__ _8_ [Don’t know]

__ _9_ [Refused ]

**Q21 What do you think was the main reason you were refused treatment or consultation?**

*INT: Present show card 20*

| __ _1_ | Was not entitled to use the service |
| --- | --- |
| __ _2_ | Long waiting time / There were no appointments |
| __ _3_ | Was discriminated against by the health professional |
| __ _4_ | The service you wanted was not provided by the public healthcare system |
| __ _5_ | Your health concern was not considered serious enough to receive treatment |
| __ _6_ | Other reason 🡪 [Q19g] What other reason?  _____________________________________________________________________ |
| __ _8_ | [Don’t know] |
| __ _9_ | [Refused] |

**Trust in physicians**

**On a scale from totally disagree to totally agree, how would you rate the following statements?**

*INT: Present show card 21*

**Q22a I trust the doctors’ judgement about my medical care.**

__ _1_ Totally disagree

__ _2_ Disagree

__ _3_ Neutral

__ _4_ Agree

__ _5_ Totally agree

__ _8_ [Don’t know]

__ _9_ [Refused

**Q22b I trust the doctors to put my medical needs above all other considerations when treating my medical problems.**

__ _1_ Totally disagree

__ _2_ Disagree

__ _3_ Neutral

__ _4_ Agree

__ _5_ Totally agree

__ _8_ [Don’t know]

__ _9_ [Refused

**Q22c** **The doctors are well qualified to manage (diagnose and treat or make an appropriate referral) medical problems like mine.**

__ _1_ Totally disagree

__ _2_ Disagree

__ _3_ Neutral

__ _4_ Agree

__ _5_ Totally agree

__ _8_ [Don’t know]

__ _9_ [Refused

**Q22d I trust the doctors to tell me if a mistake was made about my treatment.**

__ _1_ Totally disagree

__ _2_ Disagree

__ _3_ Neutral

__ _4_ Agree

__ _5_ Totally agree

__ _8_ [Don’t know]

__ _9_ [Refused

**Health literacy**

**On a scale from very easy to very difficult, how easy would you say it is to:**

*INT: Present show card 22*

**Q23a Determine when you should get a second opinion from another doctor?**

__ _4_ Very easy

__ _3_ Fairly easy

__ _2_ Fairly difficult

__ _1_ Very difficult

__ _8_ [Don’t know]

__ _9_ [Refused]

**Q23b Use information the doctor gives you to make decisions regarding your illness?**

__ _4_ Very easy

__ _3_ Fairly easy

__ _2_ Fairly difficult

__ _1_ Very difficult

__ _8_ [Don’t know]

__ _9_ [Refused]

**Q23c Find information about support services for mental health problems such as stress or depression?**

__ _4_ Very easy

__ _3_ Fairly easy

__ _2_ Fairly difficult

__ _1_ Very difficult

__ _8_ [Don’t know]

__ _9_ [Refused]

**Q23d Assess if the information on health risks in the media is reliable?**

*PROBE: TV, Internet or other media*

__ _4_ Very easy

__ _3_ Fairly easy

__ _2_ Fairly difficult

__ _1_ Very difficult

__ _8_ [Don’t know]

__ _9_ [Refused]

**Q23e Find out about activities/behaviours that are good for your mental well-being?**

*PROBE: Activities such as meditation, physical activity, going for walk, pilates*

__ _4_ Very easy

__ _3_ Fairly easy

__ _2_ Fairly difficult

__ _1_ Very difficult

__ _8_ [Don’t know]

__ _9_ [Refused]

**Q23f Understand information in the media on how you can improve your health?**

*PROBE: media includes Internet, newspapers, magazines*

__ _4_ Very easy

__ _3_ Fairly easy

__ _2_ Fairly difficult

__ _1_ Very difficult

__ _8_ [Don’t know]

__ _9_ [Refused]

| **General living conditions** |
| --- |

**We are now coming to the last part of the interview and I have a number of questions about your general living conditions.**

**Social support**

**Q24 How many people are so close to you that you can count on them if you have serious problems?**

*INT: Present show card 23*

__ _1_ None

__ _2_ 1 or 2

__ _3_ 3-5

__ _4_ 6 or more

__ _8_ [Don’t know]

__ _9_ [Refused]

**Q25 How much interest do people show in what you are doing?**

*INT: Present show card 24*

__ _5_ A lot of interest

__ _4_ Some interest

__ _3_ Neither interest nor disinterest

__ _2_ Little interest

__ _1_ No interest

__ _8_ [Don’t know]

__ _9_ [Refused]

**Q26 How easy can you get practical help from neighbours if you should need it?**

*INT: Present show card 25*

__ _5_ Very easy

__ _4_ Easy

__ _3_ Possible

__ _2_ Difficult

__ _1_ Very difficult

__ _8_ [Don’t know]

__ _9_ [Refused]

**Q26a How many adults who live in the Neustadt/Gröpelingen do you know? With *knowing* I mean: You know at least the name of the person and the person knows your name.**

*PROBE: With adults I mean persons that are at least 18 years old.*

__|__|__ *INT: Please write in the number*

**Q26b How many of these people did you see over the past 4 weeks?**

__|__|__ *INT: Please write in the number*

**Number of persons in the household**

**How many adults and children live in your household?**

**Q27a Children under 14?** __|__|__ *INT: Write in the number*

**Q27b Children 14 to 17?**  __|__|__ *INT: Write in the number*

**Q27c Adults (18+)?** __|__|__ *INT: Write in the number*

**Citizenship**

**Q28a What is your citizenship?**

*INT: Present show card 26*

__ _1_ German

*If German, please continue with* ***Q30***

__ _2_ Other

__ _3_ Dual citizenship

**Q28b**  Which other citizenship?

*INT: Use country code list*

__|__|__ *INT: Write in country code*

**Immigration status**

**Q29 What is your immigration status?**

*INT: Present show card 26*

__ _1_ Permanent residential permit

__ _2_ Temporal residential permit

__ _3_ Other (e.g. no residential permit)

__ _8_ [Don’t know]

__ _9_ [Refused]

**Country of birth**

**Q30 In which country were you born?**

*INT: If other country use country code list*

*INT: If the country does not exist anymore, ask: ‘What is the country called nowadays?’*

__ _1_ [Survey Country]

*If other please continue with* ***Q35***

__ _2_ Other 🡪 [Q30a] Which other country? __|__|__

__ _8_ [Don’t know]

__ _9_ [Refused]

**Q31 In which country was your father born?**

*INT: If other country use country code list*

*INT: If the country does not exist anymore, ask: ‘What is the country called nowadays?*

__ _1_ [Survey Country]

__ _2_ Other 🡪 [Q30a] Which other country? __|__|__

__ _8_ [Don’t know]

__ _9_ [Refused]

**Q32 In which country was your mother born?**

*INT: If other country use country code list*

*INT: If the country does not exist anymore, ask: ‘What is the country called nowadays?*

__ _1_ [Survey Country]

__ _2_ Other 🡪 [Q30a] Which other country? __|__|__

__ _8_ [Don’t know]

__ _9_ [Refused]

*If father and mother were born in another country please continue with* ***Q36***

**Q33 Was one of your grandparents born in another country, that is, outside [survey country]?**

*If no or d.k./ref. Please continue with* ***Q37***

__ _1_ Yes

__ _2_No

__ _8_ [Don’t know]

__ _9_ [Refused]

*INT: If yes:* **Which one of your grandparents was born in another country?**

*INT: Multiple answers possible. Please write in the country code. If the country does not exist anymore, ask: ‘What is the country called nowadays?*

__ _1_ [Q33a] maternal grandfather 🡪 [Q33b] Which country? __|__|__

__ _1_ [Q33c] paternal grandfather 🡪 [Q33d] Which country? __|__|__

__ _1_ [Q34a] maternal grandmother 🡪 [Q34b] Which country? __|__|__

__ _1_ [Q34c] paternal grandmother 🡪 [Q34d] Which country? __|__|__

__ _8_ [Don’t know]

__ _9_ [Refused]

*Continue with Q37*

**Year of immigration**

**Q35 In which year did you come to [survey country] to live here permanently?**

__|__|__|__ *INT: Write in year*

**Language proficiency**

**Q36a** **How would you rate your German language skills?**

*INT: Present show card 27*

__ _5_ Very good

__ _4_Good

__ _3_Fair

__ _2_ Poor

__ _1_ Very poor

__ _8_ [Don’t know]

__ _9_ [Refused]

**Q36b How would you rate your English language skills**

*INT: Still show card 27*

__ _5_ Very good

__ _4_Good

__ _3_Fair

__ _2_ Poor

__ _1_ Very poor

__ _8_ [Don’t know]

__ _9_ [Refused]

**Q36c What other languages are you proficient in?**

_____________________________________________________________________

*INT: Write in*

**Employment status**

**Q37 How would you describe your current employment status?**

*INT: Present show card 28*

__ _1_ Working for pay or profit (including unpaid work for a family business or holding, including an apprenticeship or paid traineeship, including currently not at work due to maternity, parental, sick leave or holidays)

__ _2_ Unemployed

__ _3_ Pupil, student, further training, unpaid work experience

__ _4_ In retirement or early retirement or has given up business

__ _5_ Permanently sick/ disabled

__ _6_ In compulsory military or community service

__ _7_ Fulfilling domestic tasks

__ _8_ Other 🡪 [Q37a]Which one?

__________________________________________________________________

__ _88_ [Don’t know]

__ _99_ [Refused]

**Education**

**Q38 What is the highest education leaving certificate, diploma or education degree you have obtained? Please include any vocational training.**

*INT: Present show card 29*

| __ _1_ | No degrees |
| --- | --- |
| __ _2_ | Lower secondary degree (after 9 or 10 years of schooling); no vocational certificate |
| __ _3_ | A-Level or equivalent only (no vocational or university degree); vocational certificate, apprenticeship or equivalent |
| __ _4_ | A-Level or equivalent plus vocational certificate |
| __ _5_ | Bachelor or Master degree |
| __ _6_ | Ph.D. or equivalent |
| __ _7_ | Other 🡪 [Q38a] Which one?  __________________________________________________________________ |
| __ _8_ | [Don’t know] |
| __ _9_ | [Refused] |

**Income**

**Q39 Please tell me which letter describes your household's total income from all sources, after tax and compulsory deductions? If you don't know the exact figure, please give an estimate. Use the part of the card that you know best: weekly, monthly or annual income.**

*INT: Present show card 30*

__ _1_ J

__ _2_ R

__ _3_ C

__ _4_ M

__ _5_ F

__ _6_ S

__ _7_ K

__ _8_ P

__ _9_ D

__ _10_ H

__ _88_ [Don’t know]

__ _99_ [Refused]

**You have now answered all questions. Thank you very much!**

[Int6] Time (end): __|__:__|__

[Int7] Interviewer comments:

|  |
| --- |
|  |
|  |
|  |
|  |
|  |
|  |
|  |
|  |
|  |
|  |
|  |
