# Supplementary material for: Healthcare bricolage in Europe’s superdiverse neighbourhoods: a mixed methods study
Source: BMC Public Health. 2019 Oct 22;19:1325. doi: 10.1186/s12889-019-7709-x (PMC6805362; doi:10.1186/s12889-019-7709-x)
Supplement: Supplementary file 3 — Additional file 3: Table S1. Characteristics of the comparison countries and neighbourhoods. Table S2. Qualitative Interviewee profiles. Table S3. Resources used within the types of bricolage. [file 12889_2019_7709_MOESM3_ESM.docx]

**Supplementary Table 1: Characteristics of the comparison countries and neighbourhoods**

|  | City | Health and welfare regimes |
| --- | --- | --- |
| Germany | Bremen: 10^th^ largest city  554646 residents, 30% people from migrant background (deprived and skilled) from 162 countries. | Conservative welfare regime  Universal, corporatist health care system, decentralized and self-governing. Compulsory health insurance based on income covers 85% of the population. Direct access to services with choice of provider. Migrants receive a health insurance card allowing access to medical help for acute illness, pain and pregnancy. Without insurance, people must pay or use volunteer doctors, CSOs and welfare organizations. There is no functioning interpretation system. The healthcare ecosystem is very complex so people struggle to understand entitlements. The ecosystem has been transformed into a competitive health market with statutory health insurers behaving as competing corporations. Medical professionals are supposed to report irregular migrants to immigration authorities. |
| Portugal | Lisbon: capital & largest city  547733 residents, housing migrants from 172 countries, recent arrival of refugees | Southern European welfare regime  Health system is comprise of multiple sectors including a universal national health service (NHS) with co-payment scheme and exemptions for certain populations. Health subsystems include health insurance for public servants, a growing private insurance health sector and the lottery funded charity-led parallel health service of Santa Casa da Misericordia (SCML) for vulnerable populations. The economic crisis affected provision and quality of health services as TROIKA imposed severe. Most irregular migrants’ exemptions were removed making access problematic. NHS professionals cannot report irregular migrants to authorities due to professional ethics. |
| Sweden | Uppsala: 4th largest city.  202625 residents, people from migrant background from 174 countries (deprived and skilled) | Social Democratic welfare regime  Comprehensive universal system. Equity is prioritised through redistributive policies in the form of statutory and municipal taxes, benefits and services aimed at mitigating the damaging effects of poverty. The system of fiscal and non-fiscal universal benefits, distributed with little means-testing imply extensive public-sector employment in health and social care. Healthcare and welfare available to whole population for a small fee. Only immigrants with legal rights of domicile can access non-urgent care. Very limited private sector. Provision through for-profit corporations increasing. Limited austerity since Sweden’s major financial crisis and contraction of the welfare state occurred in the 1990s. Emphasis on individual responsibility, healthy living and active lifestyles. |
| UK | Birmingham: 2^nd^ largest city.  1073045 residents,  22% foreign born, 47% ethnic minorities from 187 countries. | Liberal welfare regime  The UK's NHS introduced as a universal system with primary and secondary healthcare free to all. The past 20 years have seen constant attempts at restructuring to slow down spiraling costs. Shortages of doctors and nurses with the system said to be in crisis and Government refusing to increase the budget . Restructuring in 2013 introduced service commissioning to introduce competition, reduce costs and offer choice for health “consumers”. Widespread concerns about capacity to meet rising demand, the exacerbation of recruitment difficulties, reduced investment, long-term under-funding of mental health provision and cuts in public health and social care budgets. Immigration legislation denies undocumented migrants and failed asylum seekers free access beyond emergency care. NHS workers are expected to report and refuse to treat undocumented migrants. |

Terminology vary by country so data are not comparable.

Data for Germany: 2012 national census and Arbeitnehmerkammer: Bericht zur sozialen Lage 2013

Data for Portugal: migrant definition: foreign born and ethnic minorities

Data for Sweden: foreign born and ethnic minorities

Data for the UK: 2011 Census

**Supplementary Table 2: Qualitative Interviewee profiles**

| **Age** | **%** | **Notes** |
| --- | --- | --- |
| 18-44  45-79  80+ | 52  42    3 | Sweden and UK had over 25% of the sample over 60 years |
|  |  |  |
| **Gender** |  |  |
| women | 56 |  |
|  |  |  |
| **Civil status** |  |  |
| married | 38 | More married interviewees in German and Sweden than in UK and Portugal |
| single | 34 |  |
| separated / divorced / widowed | 26 |  |
| **Born overseas** |  | Total number of countries of birth per country |
| UK | 70 | 22 |
| Sweden | 66 | 17 |
| Germany | 63 | 16 |
| Portugal | 56 | 14 |
| **Employment** |  |  |
| employed | 49 | Economic activity highest in UK – 53%  Just under 50% elsewhere |
| unemployed | 21 | Unemployment highest in Germany at 35% & Portugal at 24% |
| retired | 11 | Most in Sweden - 17% and UK - 13% |
| **Local language** |  |  |
| Fluent / mother tongue | 30 + 35 |  |
| basic | 17 |  |
| None | 3 |  |

**Supplementary Table 3. Resources used within the types of bricolage**

| Resources used | No bricolage (n=284) | Within-system bricolage  (n=789) | Added-to-system bricolage  (n=581) | Alternative  (n=100) |
| --- | --- | --- | --- | --- |
| Public healthcare system | 100% | 100% | 100% | 0% |
| Out-of-pocket | 0% | 0% | 48.7% | 44% |
| Alternative medicine | 0% | 0% | 72.1% | 39% |
| Transnational services | 0% | 0% | 16.2% | 10% |
| Support from family and friends | 0% | 75% | 75% | 52% |
| Internet | 0% | 47.8% | 52.3% | 36% |
| Other information sources | 0% | 35.9% | 50.8% | 25% |
